# Survival Impact of an On-Site Medicalization Program in the Control of COVID-19 Outbreaks in 11 Nursing Homes

**DOI:** 10.3390/jcm12206517

**Published:** 2023-10-14

**Authors:** Bosco Baron-Franco, Manuel Ollero-Baturone, Jara Eloísa Ternero-Vega, Maria Dolores Nieto-Martín, Lourdes Moreno-Gaviño, Concepcion Conde-Guzmán, Sonia Gutiérrez-Rivero, Manuel Rincón-Gómez, Pablo Díaz-Jiménez, Juan José Muñoz-Lopez, Luis Giménez-Miranda, Celia Fernández-Nieto, Máximo Bernabeu-Wittel

**Affiliations:** 1 Internal Medicine Department, University Hospital Virgen del Rocío, 41013 Seville, Spain; 2 Internal Medicine Department, Hospital Alta Resolución de Utrera, 41710 Seville, Spain; 3 Department of Medicine, University of Seville, 41004 Seville, Spain

**Keywords:** COVID-19, nursing homes, primary care, hospitals, medicalization, palliative care, aged, survival

## Abstract

Background: The elderly admitted to nursing homes have especially suffered the havoc of the COVID-19 pandemic since most of them are not prepared to face such health problems. Methods: An innovative coordinated on-site medicalization program (MP) in response to a sizeable COVID-19 outbreak in three consecutive waves was deployed, sharing coordination and resources among primary care, the referral hospital, and the eleven residences. The objectives were providing the best possible medical care to residents in their environment, avoiding dehumanization and loneliness of hospital admission, and reducing the saturation of hospitals and the risk of spreading the infection. The main outcomes were a composite endpoint of survival or optimal palliative care (SOPC), survival, and referral to the hospital. Results: 587 of 1199 (49%) residents were infected, of whom 123 (21%) died. Patients diagnosed before the start of the MP presented SOPC, survival, and referrals to the hospital of 83%, 74%, and 22.4%, opposite to 96%, 84%, and 10.6% of patients diagnosed while the MP was set up. The SOPC was independently associated with an MP (OR 3.4 [1.6–7.2]). Conclusion: During the COVID-19 outbreak, a coordinated MP successfully obtained a better rate of SOPC while simultaneously reducing the need for hospital admissions, combining optimal medical management with a more compassionate and humanistic approach in older people.

## 1. Introduction

Coronavirus 2019 disease (COVID-19) is caused by the severe acute respiratory syndrome coronavirus 2 (SARS-CoV-2). It was first described in December 2019 in Wuhan, China, and since then, it has been plaguing the world population, testing the healthcare systems of all countries. Three years after its onset, in December 2022, there were over 656 million confirmed cases and over 6.6 million deaths reported worldwide [1]. Spain is one of the countries that has been most severely affected by this disease, presenting high rates of infection, hospitalization, and COVID-19-related deaths. As of the end of December 2022, Spain recorded a 14-day notification rate of over 4000 cases per 100,000 population and more than 117,000 deaths [2].

SARS-CoV-2 infection is more severe in older people, with a higher proportion of severe illness and death compared to non-elderly adults or young individuals. Although the appearance of SARS-CoV-2 vaccines has greatly mitigated the impact of the disease, during the first three waves of the pandemic, the effect was devastating since they were not available. While young infected people only have a 19.8% chance of developing severe complications from COVID-19, the probability rises to 80.7% for individuals 80 years and older [3]. Recurring evidence indicates that being older and having underlying chronic diseases are significant risk factors for COVID-19 [4] and suggests that they are the two most important factors in predicting a worse clinical outcome. Older people suffer a worse evolution due to COVID-19. Multiple factors have been proposed, such as deficiencies in the social and healthcare systems, a shortage of material and personnel, difficult access to medical care, etc. [5,6].

The development of the pandemic is especially devastating in residences, where numerous situations are combined [7]. First, the nursing home population has an average age in the 80s, most having multiple comorbidities and/or immunocompromised states that predispose them to severe forms of COVID-19 with higher mortality rates [8]. Second, they may have underestimated the true prevalence in nursing facilities. Lack of viral testing capability, difficulty in performing nasal swab tests in the cognitively impaired population, and difficulty in recognizing symptoms of COVID-19 in the nursing home population because they used non-typical tests could explain this issue [9]. Third, the transmission of the virus is easier (lack of social distancing, difficulty in wearing a mask when cognitively impaired, etc.) [10]. Lastly, many nursing homes face material shortages; their facilities are not prepared for the isolation requirements in the event of a pandemic, they are not well connected with the health system, and the staff’s low numbers and limited training in this new situation does not help to improve the impact of it [11]. Thus, although data are scarce, numerous outbreaks have been declared in nursing homes that have affected residents, working staff, and resident’s families [12].

This paper will provide a comprehensive description of a novel on-site medicalization program (MP) implemented in response to a significant three-wave COVID-19 outbreak. These outbreaks affected eleven nursing homes in the province of Sevilla, located in Southern Spain. The province has a total population of 1,942,389 inhabitants, with 688,592 of them living in the city. The MP facilitated a collaborative and coordinated effort among the staff, the resources of primary care, the referral hospital, and the nursing home residences.

This program was designed to deliver the highest quality medical care to residents infected with COVID-19 within their familiar environment. This approach aimed to prevent the dehumanization and isolation often associated with hospital admissions while also helping to alleviate the strain on hospitals and reduce the risk of community transmission. Specifically, we will describe the MP and its outcomes during the three waves, with a focus on survival rates and the provision of optimal palliative care to residents.

## 2. Patients and Methods

In March 2020, patients from a nursing home in our area began to arrive at the emergency department of our hospital, followed within a few days by other elderly people from other residences. This motivated the decision to create an integrated care plan for nursing home residents, coordinated between the hospital, primary care, and the staff of each residence, transferring human and material resources to the residences so that the residents could receive the treatment appropriate to their level of needs, which could be anything from oxygen therapy and the medical treatment for COVID-19 used at the time to appropriate palliative care when necessary.

The objectives of this MP were as follows: (1) To achieve care for residents with adequate resources, similar to what is offered at the hospital, but maintaining humanized care, allowing residents to remain in their environment with their colleagues and nursing home staff, avoiding the isolation and depersonalization that hospitalization entails in patients infected with COVID-19. (2) To avoid hospital saturation by reducing the use of beds for COVID-19 admissions, allowing the admission of other patients, and making the hospital collapse harder. (3) To decrease the risk of spreading the virus by keeping residents in the same place.

The MP included the following: transferring the knowledge, protocols, circuits, and personnel from the hospital to the residences; providing the residences with medical resources and materials that allow them to handle most patients; and training the residence staff. For this, we were convinced that care for outbreaks in the nursing homes was a responsibility of the Internal Medicine service, shared with other actors (mainly primary care), and we believed we could guarantee clinical care in the residences equivalent to that administered in the hospital.

## 3. Reference Population and Inclusion Criteria

Every resident who tested positive for COVID-19 was enrolled in the MP. The MP was implemented during the first wave in NH4 (March and April 2020), during the second wave in NH6 (September to November 2020), and during the third wave in NH1 (January 2021).

## 4. Medicalization Program Characterization

1. The areas of the residence were divided into clean and contaminated areas. A “clean area” with common areas and rooms for uninfected residents and a contaminated area also with rooms and common areas where affected residents were transferred were established. All members of the work team were required to wear personal protective equipment when staying within the contaminated area.

Within the clean zone, an area for clinical work, administrative tasks, and computer equipment was located, with healthcare electronic information systems, as well as a secure lock room for healthcare workers to dress and undress.

2. Diagnostic testing during the first wave was performed using a SARS-CoV-2 real-time reverse transcription polymerase chain reaction (rRT-PCR) panel for the detection of SARS-CoV-2 and a lateral flow serologic technique with fingerstick blood samples for the identification of specific antibodies against SARS-CoV-2. During the second wave, a diagnostic test was performed using a SARS-CoV-2 real-time reverse transcription polymerase chain reaction (rRT-PCR) panel, lateral flow immunoassay SARS-CoV-2 antigen detection test, or both for the detection of SARS-CoV-2.

During the first wave, a confirmed case was defined as a person present in the nursing home with a positive SARS-CoV-2 RT-PCR on a nasopharyngeal sample, the detection of either IgM or IgM and IgG antibodies (referred to as “positive serology”), or both. During the second and third waves, a confirmed case was defined as a person present in the nursing home with a positive SARS-CoV-2 RT-PCR on a nasopharyngeal sample or a positive lateral flow immunoassay SARS-CoV-2 antigen detection test.

3. The healthcare worker provision involved a round-the-clock clinical care service provided by mixed teams of physicians and nurses, covering all seven days of the week. These teams were composed of healthcare professionals from primary care, the hospital’s Internal Medicine Department, and emergency departments. A total of 60 healthcare workers were mobilized for this effort, consisting of 35 physicians and 25 nurses. They underwent specialized training for the management and treatment of COVID-19 patients. To ensure safety, a clear division was maintained between personnel working in the clean area and those operating in the contaminated area. Adequate clinical care was provided, and any staff members who tested positive for acute SARS-CoV-2 infection were promptly isolated and quarantined until they received a negative result from a weekly nasopharyngeal swab PCR test, starting from the first day they exhibited no symptoms.
4. A standardized clinical management and treatment algorithm was developed, along with a uniform communication protocol to provide daily updates to relatives via phone regarding the clinical condition of both affected and unaffected residents. Electronic admission was implemented for all residents with confirmed SARS-CoV-2 infection. This electronic system enabled healthcare providers to access and update electronic health records, request blood extractions, and prescribe medications as if the patients were being treated within a hospital setting.
5. A thorough epidemiological investigation was conducted, including continuous monitoring to trace the origin and progression of the outbreaks. Additionally, there was an adequate supply of essential equipment, disposable items, and medications. This supply encompassed materials for blood extractions, intravenous and subcutaneous lines, intravenous fluids, oxygen therapy equipment, electrocardiographs, a portable ultrasound machine, and various hospital medications, such as antiviral agents and intravenous drugs, among other essential provisions.

## 5. Clinical Algorithm and Treatment Protocols

The team that cared for each resident with COVID-19 infection carried out an exhaustive, comprehensive multimodal assessment (clinical, functional, psycho-affective, social, pharmacological, etc.), also considering the patient and their relatives’ preferences and consulting the patient’s advance directives for life support. On this basis, either active standard care or advanced palliative care were offered. Patients were assessed daily, including holidays, and health personnel were present 24 h a day for urgent care.

### 5.1. Active Standard Care

The treatment offered to the residents in this group was similar to that of the patients admitted to the hospital in terms of drug treatment, oxygen supply, blood tests, lung ultrasound, etc. In the event of worsening, referral to the hospital was offered for more aggressive medical management, including increased oxygen supply with a nasal cannula or even admission to the intensive care unit if necessary. The available antiviral treatment was the one for which the most scientific evidence was available at the time (in the first wave, hydroxychloroquine, lopinavir/ritonavir, and azithromycin, and later steroids, low molecular weight heparin, remdesivir, and tocilizumab).

### 5.2. Advanced Palliative Care

Personalized care, prioritized comfort, spiritual support, and individual rooms were provided to patients in this group. A comprehensive multidimensional assessment was conducted, prioritizing symptom control, and medications were adjusted daily as needed. Clinicians were proactive in identifying and addressing end-of-life and distressing symptoms as they arose. In such cases, patients were offered palliative sedation, and one family member was allowed to accompany the patient while following specific instructions for using personal protective equipment. The criteria for optimal palliative care were met when the entire process, including offering, acceptance, and implementation of the aforementioned measures and the option of palliative sedation and accompaniment when deemed necessary, was successfully carried out.

### 5.3. Medical Monitoring, Referrals, and Medical Discharge

Every patient received daily medical supervision until their symptoms disappeared. In the context of this study, a 30-day timeframe was defined following confirmation of SARS-CoV-2 infection. Hospital referrals were arranged and scheduled by the clinicians overseeing in-hospital COVID-19 cases when deemed clinically necessary. All patients remained in isolation within the “contaminated area” for 14 days after becoming asymptomatic. Subsequently, a follow-up nasopharyngeal swab PCR test was conducted prior to their discharge and transfer to the “clean area”.6. Data Collection and Variables

We retrospectively registered a priori selected factors, including demographic details, clinical information, functional assessments, and pharmacological data, for all the patients included in our retrospective analysis. These factors encompassed demographic characteristics, any existing comorbidities, prior treatments, assessment of physical function using the Barthel index [13], prognosis as measured by the PROFUND index [14], COVID-19 symptoms and signs at the time of presentation, medical treatments administered during the outbreak, and the ultimate patient outcomes.

The main outcome was a composite variable, which was accomplished if survival or optimal palliative care (SOPC) of residents with COVID-19 occurred. For this purpose, we looked at survival as dichotomous, so subjects were categorized depending on whether they survived COVID-19 or not after the follow-up period. Optimal palliative care was defined as detailed previously.

Secondary outcomes were survival in those patients stratified to active standard care and the number of patients who needed to be referred to the hospital. To achieve this objective, we examined survival in two ways: as a binary outcome and as a time-dependent variable. In the binary outcome analysis, individuals were categorized based on whether they survived COVID-19 during the follow-up period or not. We also registered differences in outcomes between the first, second, and third waves.

## 6. Statistical Analysis

The dichotomous variables were presented using whole numbers and percentages, while continuous variables were expressed as either mean and standard deviation or median and interquartile range in cases where they did not follow a normal distribution. The distribution of all variables was assessed using the Kolmogorov–Smirnov test. To explore potential differences in SOPC, survival and patients needing hospital referral were investigated first, and we initially employed the chi-squared test with Yates correction and, when necessary, the Fisher exact test for dichotomous variables. For normally distributed quantitative variables, Student’s t-test was used, while the Mann–Whitney U test was applied for quantitative variables that did not follow a normal distribution.

Factors that exhibited statistically significant differences in the unadjusted analysis were included in a multivariable backward stepwise logistic regression model to identify those independently associated with SOPC. The strength of these associations was quantified using odds ratios (OR) along with 95% confidence intervals. The statistical analysis was conducted with SPSS 22.0 software.

## 7. Ethical Aspects

Anonymous clinical data use was provided by all the patients or their legal representatives for the investigation. This study received approval from the local ethics committee under internal code 1199-N-20. In the context of this retrospective project, the collection, processing, and analysis of all data were conducted anonymously, exclusively for the purposes of the project. All data were safeguarded in compliance with the European Union directive 2016/679 of the European Parliament and the European Council, dated 27 April 2016, pertaining to the protection of individuals and their personal data.

## 8. Results

The total population was 1199 residents, 587 of whom were infected (86 years old (Quartile 1–Quartile 3 (Q1–Q3) 79–90 years old), 77% women). The MP was implemented in 11 nursing homes; 1 of them had two outbreaks (NH3 and NH11). A comprehensive overview of the extent of the outbreak is provided in Table 1. Out of a total of 1199 residents, 587 (49%) were infected by SARS-CoV-2; 123 of them (21%) died (Table 1). A total of 272 were infected during the first wave, and 315 were infected during the second and third wave. Eleven days [7,8,9,10,11,12,13,14] were the median between the diagnosis of the first case of COVID-19 and when the MP was initiated. Furthermore, the median length of time the MP was in effect was 39 days [37 to 41].

Table 2 provides a comprehensive breakdown of the main clinical and biological characteristics of all affected residents, along with the most prevalent complications. In a summarized way, they were mostly aged women with multiple chronic conditions, severe dependence on assistance in daily living activities, a high-intermediate one-year mortality risk, and polypharmacy. The primary symptoms reported included fatigue and overall decline in health, dyspnea, cough, mild fever, loss of appetite, and episodes of delirium. One hundred and seventy-three residents (29.5%) stayed asymptomatic during the infection. The most frequent symptoms and main biological parameters are described in Supplementary Table S1.

Residents affected by COVID-19 were diagnosed with a positive nasopharyngeal swab PCR test alone (440 (75%)), positive nasopharyngeal antigen test alone (78 (13.3%)), PCR test and rapid serological test (29 (5%)), serological test alone (22 (3.7%)), and PCR and antigen test (17 (3%)). The active medical standard treatment was proposed for 370 (63%) patients, and the advanced palliative care approach was proposed for 217 (37%) after a first evaluation. A summary of treatments used in residents with COVID-19 infection is described in Table 3.

The main outcomes are detailed in Table 4. A total of 217 patients were identified for management with palliative advanced care; 52 (24%) received palliative sedation, and all of them died. A total of 10% (*n* = 37) and 39.6% (*n* = 86) of patients proposed for active standard or optimal palliative (SOPC) care died, respectively. Likewise, those patients who were treated during the MP had a better evolution with fewer complications, fewer referrals to the hospital, longer survival, and higher SOPC.

In Table 5, we describe the symptoms and biological parameters associated with survival. A total of 97 (16.5%) of the infected patients were transferred from their nursing homes. A total of 81 of them went to their referral hospital, and 16 went to two medicalized hotels transformed into COVID-19 hospitals during the first wave.

Multivariate analysis results are described in Table 6.

## 9. Discussion

The world has recently been hit by a SARS-CoV-2 pandemic that generated a health crisis, exceeding the response capacity of all health systems. It particularly affected the most vulnerable people, such as the elderly and institutionalized patients. The implementation of an MP as part of the strategy to manage 12 COVID-19 outbreaks across 11 nursing homes in Seville, Spain, led to a significant improvement in the combined endpoint of survival or optimal palliative care (SOPC). Additionally, it contributed to a reduction in the rates of hospital referrals. This program brought together professionals from various medical specialties, including Internal Medicine, primary care, and the emergency department, while also utilizing resources from both the hospital and primary care settings.

Half of the residents were infected (49%), presenting a mortality before the MP of 26%, like the majority of published studies, which was around 30 and even above 35% in some cases [15,16,17,18,19,20]. Similarly, overall mortality in Spain in this period in patients older than 80 years was 30.6% [21]. However, the establishment of the MP led to a significant reduction in mortality of up to 16%. This indicates the success of joint and multidisciplinary work when addressing the care of complex patients. Additionally, the MP allowed fewer complications and referrals to the hospital, as well as better care in the event of poor life expectancy.

The key factors contributing to the success of the MP include the following: 1. Early recognition of symptoms and management of complex patients by trained medical personnel, which allows anticipating complications and acting before they appear. 2. The management of sick residents in limited areas, which allowed early mobilization and maintaining the relationship with the environment without the strict isolation of hospitals, greatly reducing the risk of the appearance of some geriatric syndromes such as immobility, delirium, etc. 3. Being able to apply more humane palliative care for patients and their families, allowing residents to be assisted in their environment, without giving up adequate treatment for COVID-19. In fact, 60.4% of the patients included in the palliative care program survived.

On the other hand, in addition to the influence of the MP on mortality, it is interesting to note that no isolated comorbidity modified the prognosis. However, their union in the PROFUND prognostic index did [22,23,24,25,26,27]. In addition, mortality was related to age, as expected, and it was also linked to a lower Barthel index. This suggests that these indices may be useful when predicting the prognosis and making medical management decisions. Furthermore, some symptoms, such as dyspnea, delirium, or global deterioration, along with some analytical parameters, such as increased CRP or ferritin, were associated with increased mortality. Therefore, healthcare providers should remain vigilant when dealing with elderly patients exhibiting these signs.

This study allowed us to obtain a broad vision of the characteristics of the institutionalized population in residences infected with COVID-19. It reveals some relevant differences with respect to the general population. The majority were women (75%), which is consistent with the findings in most studies [28,29,30,31]. The infected patients in our series presented a high number of chronic pathologies, marked functional deterioration (Barthel index below 50), a high percentage of polypharmacy, and a medium–high probability of dying within a year. This aligns with the description in the majority of articles published so far, where a high percentage of patients had comorbidities such as hypertension, dementia, and chronic heart or lung diseases [28].

The presentation of the disease caused by SARS-CoV-2 in this group of patients is usually atypical and even asymptomatic [28,32,33,34,35]. This may be due to the fact that the characteristics of the patients in residences (advanced age with immunosenescence or presence of comorbidities) could influence the form of presentation of the disease. Unlike younger people, cold symptoms or anosmia are rarer in this population, and in nursing homes, it is more common for general deterioration, dyspnea, cough, low-grade fever, or anorexia, as well as even digestive symptoms, to appear [36,37]. Clinicians caring for older patients with COVID-19 should be aware of this constellation of “atypical” symptoms to expedite the diagnosis and treatment initiation, which can be particularly crucial.

Our study agrees with others, indicating that the most frequent symptoms were fatigue and global deterioration, dyspnea, cough, fever, anorexia, and delirium. These symptoms were also more strongly associated with higher mortality, consistent with previously published findings [36,37]. On the other hand, coinciding with other publications, approximately a third of the elderly infected in residences remained asymptomatic, which confirms that screening for infected people should be carried out periodically since there is a high percentage of asymptomatic patients who transmit the infection.

### What Can We Say That We Have Learned from the MP?

1. The importance of nursing homes in the health system, which, although they are on the border between health and social care, are key elements that must be addressed with an adequate response from the health system, especially in the face of serious threats such as the case of a pandemic like the one we have experienced.
2. The health system must learn to redistribute material and human resources according to the needs of the population, moving from hospital immobility and learning to go out when necessary, adapting to the requirements of the moment and of the population. One of the functions and objectives of the medicalization of the residences has been to avoid the further collapsing of hospital resources, since they were already in a precarious situation. The purpose was to treat patients (as far as possible) within the care homes themselves and transfer only the really necessary cases. Thus, several hundred patients would have been referred to the hospital in this period if the residences had not been medicalized, with the consequent risk of collapse of the emergency department and hospitalization wards, which were already overwhelmed.
3. Collaborative efforts between primary care and the hospital are far more potent than the sum of the work of both separately. Although there are already experiences where both levels work synchronously, despite the difficulties, these tend to be isolated experiences that start from the professionals themselves who join forces and improve patient care. However, this MP project shows that in situations of maximum difficulty and lack of resources, joint work between primary care and hospitals is the best and most efficient way to respond to a crisis that threatens an entire health system due to a global pandemic.

There are some limitations in this investigation that we should point out. First, as it is a retrospective study, it is possible that some bias could have been introduced in the data collection. Secondly, not all the residences of this population were included, but only those that presented outbreaks, so the population might not be faithfully represented, although we think that, as there are a high number of residents in this study, it is unlikely that there will be significant differences between the population and the sample. Lastly, there could be the phenomenon of regression to the mean, which often occurs in studies carried out on high-risk populations; however, if it exists, we believe that it is compensated because all those patients who started the symptoms before the MP but who were treated by the program were treated in the data analysis as if they had not been treated by the MP.

In conclusion, we believe that the implementation of a residence medicalization program, shared between hospital and primary care, is a model that improves the response to a pandemic like the one we have experienced. A rapid implementation protocol should be included in the arsenal of responses for the health systems facing a pandemic.

## Figures and Tables

**Table 1:** Global data of the 12 COVID-19 outbreaks in 11 nursing homes in the city of Seville, Spain.

	Wave	Number of Residents	Number of Affected Residents ^a^	Age of Affected Residents ^b^	Gender of Affected Residents (Female)	Number of Deaths in Affected Residents ^c^	Date of Outbreak Start	Days Until MP Started
NH1	1	168	123 (73.2%)	89 (83–91)	93 (76%)	29 (23.6%)	17 March 2020	15
NH2	1	155	93 (60.0%)	84 (78–88)	69 (44.5%)	23 (24.7%)	23 March 2020	11
NH3	1	101	35 (34.7%)	89 (84–93)	28 (80%)	6 (17.1%)	25 March 2020	12
NH4	1	33	21 (63.6%)	89 (83–90)	15 (45%)	3 (14.3%)	26 March 2020	11
NH5	2	149	103 (69.1%)	84 (78–89)	65 (44%)	21 (20.4%)	6 September 2020	10
NH6	2	77	58 (75.3%)	86 (82–99)	53 (68%)	10 (17.2%)	7 September 2020	11
NH7	2	118	50 (42.4%)	80 (75–87)	50 (100%)	3 (6.0%)	2 October 2020	6
NH8	2	69	11 (15.9%)	88 (83–91)	6 (54.5%)	2 (18.2%)	6 October 2020	21
NH9	2	130	33 (25.4%)	83 (71–89)	24 (73%)	5 (15.2%)	7 October 2020	19
NH10	2	57	32 (56.1%)	86 (82–90)	27 (84%)	14 (43.8%)	1 November 2020	3
NH11	2	104	11 (10.6%)	82 (79–90)	7 (64%)	2 (18.2%)	3 November 2020	11
NH12	3	38	17 (44.7%)	87 (84–92)	17 (100%)	5 (29.4%)	25 January 2021	1
TOTAL		1199	587 (49%)	86 (79–90)	454 (77%)	123 (21.0%)		

NH, nursing home. MP, medical program; ^a^ With respect to all residents. ^b^ Median [Quartile 1–Quartile 3]. ^c^ With respect to all affected with COVID-19 (lethality).

**Table 2:** Key clinical characteristics and complications observed in residents with COVID-19 across 11 nursing home outbreaks in Seville, Spain.

Clinical Characteristics	Global (*N* = 587)	Residents with COVID-19 Diagnosed before MP (*N* = 295)	Residents with COVID-19 Diagnosed during MP (*N* = 292)	*p*
Mean (SD)/Median [Q1–Q3]/No. (%)				
Age	86 (79–90)	86 (80–91)	85 (78–90)	0.124
Female gender	454 (77.3%)	218 (73.9%)	236 (80.8%)	0.049
Comorbidities per patient	4 (3–6)	4 (3–6)	4 (3–6)	0.859
Principal comorbidities				
Hypertension	432 (73.7%)	223 (75.9%)	209 (71.6%)	0.24
Dyslipidemia	224 (38.2%)	115 (39%)	109 (37.3%)	0.68
Advanced dementia	220 (37.5%)	111 (37.6%)	109 (37.3%)	0.94
Osteoarthritis causing affected BADL	171 (29%)	78 (26.4%)	93 (31.8%)	0.149
Diabetes mellitus	149 (25.4%)	75 (25.4%)	74 (25.3%)	0.982
Mild–moderate dementia	130 (22.1%)	67 (22.8%)	63 (21.6%)	0.724
NL disease with severe impairment	130 (22.1%)	52 (17.6%)	78 (26.7%)	0.008
Depression	117 (19.9%)	63 (21.4%)	54 (18.5%)	0.385
Atrial fibrillation	98 (16.7%)	53 (18.0%)	45 (15.4%)	0.407
Cerebrovascular disease	92 (15.7%)	47 (15.9%)	45 (15.4%)	0.862
Chronic heart failure	73 (12.4%)	39 (13.2%)	34 (11.6%)	0.563
COPD or asthma	63 (10.7%)	34 (11.5%)	29 (9.9%)	0.533
Obesity	65 (11.1%)	32 (10.8%)	33 (11.3%)	0.861
Hypothyroidism	63 (10.7%)	28 (9.5%)	35 (12%)	0.322
Anxiety disorders	62 (10.6%)	24 (8.1%)	38 (13%)	0.055
Coronary artery disease	55 (9.4%)	38 (12.9%)	17 (5.8%)	0.003
Parkinson disease	49 (8.3%)	27 (9.2%)	22 (7.5%)	0.478
Advanced chronic kidney disease	40 (6.8%)	22 (7.5%)	18 (6.2%)	0.534
Basal Barthel’s index	48.8 (32.5)	47.8 (31.5)	49.7 (33.3)	0.503
PROFUND index	7.9 (4.2)	8.1 (4.5)	7.8 (3.9)	0.376
No. of chronically prescribed drugs	7.2 (3.8)	7.0 (3.8)	7.4 (3.8)	0.261
Patients with extreme polypharmacy (>10 drugs)	118 (20.1%)	55 (18.7%)	63 (21.6%)	0.375
Patient with complications	252 (43%)	147 (50%)	105 (36%)	0.001
Acute respiratory failure	225 (38%)	132 (45%)	93 (32%)	0.001
LRT bacterial infections	81 (14%)	37 (12.8%)	44 (15.2%)	0.411
Persistent or incidental delirium	79 (13.5%)	45 (15.5%)	34 (11.7%)	0.183
Immobility and “bedridden syndrome”	75 (13%)	40 (13.8%)	35 (12.1%)	0.536
Acute renal failure	35 (6%)	22 (7.6%)	13 (4.5%)	0.117
Oropharyngeal dysphagia	30 (5%)	16 (5.6%)	14 (4.8%)	0.686
Urinary tract infection	26 (4.4%)	12 (4.1%)	14 (4.8%)	0.682
Pressure ulcers	24 (4.1%)	13 (4.5%)	11 (3.8%)	0.677
Number of complications per patient	1.04 (1.5)	1.24 (1.6)	0.8 (1.3)	0.019

Note: ALAT = alanine aminotransferase; ASAT = aspartate aminotransferase; CRP = C-reactive protein; BADL = basic activities of daily living; COPD = chronic obstructive pulmonary disease; MP = medicalization program; NL = neurological; LRT = low respiratory tract; SD = standard deviation; and Q1–Q3 = Quartile 1–Quartile 3. Bold values refer to those with significant differences in patients diagnosed before vs. during MP. Significance threshold was 0.05.

**Table 3:** Summary of treatment used in residents.

Parameter, No. (%)	Global (*N* = 587)	First Wave (*N* = 272)	Second and Third Waves (*N* = 315)
Most frequently performed medical actions			
Parenteral drugs	317 (54%)	124 (46%)	193 (61%)
Oxygen therapy	257 (44%)	114 (42%)	143 (45%)
Intravenous lines and fluids	180 (31%)	86 (32%)	94 (30%)
Point-of-care ultrasound	50 (8.5%)	22 (8%)	28 (9%)
Transfusion of blood products	2 (0.3%)	0	2 (0.3%)
Most frequent treatment administered			
Low-molecular-weight heparin	305 (52%)	118 (43%)	187 (59%)
Antimicrobials	159 (27%)	69 (25%)	90 (29%)
Corticosteroids	172 (29%)	55 (20%)	117 (37%)
Patients proposed for active standard care			
Antiviral treatment	261 (44%)	139 (51%)	187 (59%)
FIRST WAVE			
Hydroxychloroquine (HCQ)		109 (78%)	
HCQ plus Lopinavir/Ritonavir (LPV/RTV)		18 (13%)	
HCQ plus azithromycin		11 (8%)	
SECOND WAVE			
Low-molecular-weight heparin			187 (59%)
Dexamethasone			109 (19%)
Dexamethasone plus remdesivir			8 (1.4%)
Remdesivir			5 (1%)

**Table 4:** COVID-19 main outcomes.

Parameter, Mean (SD)/Median [Q1–Q3]/No. (%)		Global (*N* = 587)	Residents with COVID-19 Diagnosed before MP (*N* = 295)	Residents with COVID-19 Diagnosed during MP (*N* = 292)	*p*
Outcomes					
	Composite endpoint *	525 (89.4%)	245 (83%)	280 (96%)	<0.001
	Survival	464 (79%)	218 (74%)	246 (84%)	0.002
	Patients transferred to hospital	97 (16.5%)	66 (22.4%)	31 (10.6%)	<0.001

Note: MP = medicalization program; SD = standard deviation; and Q1–Q3 = Quartile 1–Quartile 3. Bold values refer to those with significant differences in patients diagnosed before vs. during MP. Significance threshold was 0.05. * Composite endpoint of survival or optimal end-of-life care.

**Table 5:** Symptoms and biological parameters associated with survival.

		Patients That Survived			
Mean (SD)/Median [Q1-Q3]/No. (%)					
		Yes	No	*p*	Odds Ratio, IC
Age		87 (11)	89 (8)	<0.001	1.066 (1.037–1.096)
Basal Barthel Index		60 (50)	35 (48)	<0.001	0.974 (0.966–0.982)
Profund scale		7 (6)	9 (4)	<0.001	1.152 (1.094–1.214)
Presence of symptoms		295 (71.3%)	169 (97.1%)	<0.001	1.509 (1.397–1.631)
	Fever	150 (65%)	312 (88%)	<0.001	2.012 (1.674–2.418)
	Cough	104 (62.3%)	358 (85.4%)	<0.001	2.257 (1.772–2.875)
	Dyspnea	108 (53%)	354 (92.4%)	<0.001	3.277 (2.706–3.969)
	Anorexia	79 (61%)	383 (84%)	<0.001	2.358 (1.758–3.163)
	Delirium	50 (46.3%)	413 (86.4%)	<0.001	4.367 (3.165–6.024)
	Global deterioration	130 (57%)	333 (93%)	<0.001	2.843 (2.398–3.372)
Main biological parameters					
	Lymphocytes (no./µL)	1263.3 (162.3)	1190 (712.3)	0004	0.999 (0.998–1.000)
	CRP	67.2 (53.7)	103.5 (80)	<0.001	1.014 (1.010–1.019)
	Ferritin (ng/mL)	456.4 (161.4)	639 (413.5)	<0.001	1.001 (1.001–1.002)
	Leukocytes (no./µL)	7968 (2309.5)	9169.1 (4782.4)	0.02	1.000 (1.000–1.000)
	Creatinine (mg/dL)	1.3 (0.47)	1.36 (1.1)	0.028	1.311 (1.013–1.694)
	ASAT	26.1 (7.8)	40 (26.2)	<0.001	1.046 (1.029–1.064)
	ALAT	21.2 (6.8)	32.8 (26.2)	<0.001	1.047 (1.028–1.067)
	Creatinine kinase	116.3 (70.4)	159.5 (122)	<0.001	1.006 (1.003–1.008)

Note: ALAT = alanine aminotransferase; ASAT = aspartate aminotransferase; CRP = C-reactive protein; SD = standard deviation; and Q1–Q3 = Quartile 1–Quartile 3. Significance threshold was 0.05.

**Table 6:** Multivariable logistic regression analyses to explore the association between prognostic factors and composite endpoint of survival or optimal end-of-life care (SOPC) in nursing home residents with COVID-19.

Composite Endpoint of Survival or Optimal End-of-Life Care (SOPC)				
Prognostic Factors		Adjusted Or	CI	*p* Value
	Lack of cerebrovascular disease comorbidity	3.9	1.2–12.1	0.02
	Inclusion in MP	3.4	1.6–7.2	0.001
	Absence of dyspnea	3.1	1.3–7	0.008
	Better PROFUND index	0.94	0.9–1.0	0.05
	Lack of complications	0.93	10–836	<0.001
	Receiving low-molecular-weight heparin	0.27	0.13–0.55	<0.001

Note: MP = medicalization program. OR = odds ratio. Significance threshold was 0.05.

## Data Availability

Data regarding this study are available upon request to the corresponding author.

## Supplementary Materials

The following supporting information can be downloaded at: https://www.mdpi.com/article/10.3390/jcm12206517/s1, Figure S1: Distribution of residents in all the nursing homes; Table S1: Most frequent symptoms and main biological parameters of residents with COVID-19 during 11 nursing home outbreaks in Sevilla, Spain.
