# Supplementary material for: Survival Impact of an On-Site Medicalization Program in the Control of COVID-19 Outbreaks in 11 Nursing Homes
Source: J Clin Med. 2023 Oct 14;12(20):6517. doi: 10.3390/jcm12206517 (PMC10607111; doi:10.3390/jcm12206517)
Supplement: Supplementary file 1 [file jcm-12-06517-s001.zip › jcm-2618749-supplementary.pdf]

Supplementary Table S1: Most frequent symptoms and main biological parameters of Residents With COVID-19 During 11 Nursing Home Outbreaks in Sevilla, Spain

Clinical Features<br>Mean (SD) / Median [Q1-Q3] / No. (%)	Global (N = 587)	Patients Diagnosed With COVID-19 Before MP (N = 295)	Patients Diagnosed With COVID-19 During MP (N = 292)	p
Most frequent symptoms				
Fatigue and global deterioration	229 (39%)	125 (42.5%)	104 (35.6%)	0.087
Dyspnea	203 (34.6%)	128 (43.5%)	75 (25.8%)	**<0.001**
Cough	167 (28.4%)	85 (29%)	82 (28.1%)	0.804
Low grade fever (37-37.9º C)	158 (27%)	91 (31%)	67 (22.9%)	0.063
Anorexia	129 (22%)	62 (21.1%)	67 (23%)	0.572
Delirium	108 (18.4%)	62 (21.2%)	46 (15.8%)	0.092
Diarrhea	76 (12.9%)	36 (12.2%)	40 (13.7%)	0.600
High grade fever (≥38º C)	73 (12.4%)	38 (12.9%)	35 (12.1%)	0.051
Nausea/Vomiting	42 (7.2%) / 32 (5.5%)	20 (6.8%) / 13 (4.4%)	22 (7.5%) / 19 (6.5%)	0.731/0.267
Rhinorrhea	21 (3.6%)	13 (4.4%)	8 (2.7%)	0.273
Odynophagia	19 (3.2%)	11 (3.7%)	8 (2.7%)	0.494
Falls	19 (3.2%)	10 (3.4%)	9 (3.1%)	0.827
Headache	18 (3.1%)	8 (2.7%)	10 (3.4%)	0.622
Ageusia	14 (2.4%)	11 (3.9%)	3 (1.1%)	**0.031**
Anosmia	10 (1.7%)	8 (2.8%)	2 (0.7%)	0.056
Asymptomatic	173 (29.5%)	73 (24.7%)	100 (34.2%)	**0.012**
Main biological parameters				
Hemoglobin (g/dL)	12.2 (2.2)	12.3 (2.3)	12.0 (2.1)	0.955
Leukocytes (no./µL)	7582 (4181)	8431 (4532)	6849 (3716)	**0.003**
Lymphocytes (no./µL)	1271 (684)	1189 (691)	1343 (673)	0.071
Platelets (no./µL)	231 341 (103 320)	236 757 (112 042)	226 705 (95 391)	0.443
D Dimer	2571 (6292)	2638 (5833)	2515 (6675)	0.886
Creatinine (mg/dL)	1.14 (1.0)	1.23 (1.0)	1.06 (1.0)	0.187
ASAT	30 (24.9)	34 (27)	27 (22)	0.051
ALAT	23 (21.8)	27 (28)	20 (14)	**0.021**
Creatinine kinase	135 (323)	158 (250)	117 (374)	0.329
CRP	64 (88)	83 (92)	48 (81)	**0.002**
Ferritin (ng/mL)	430 (623)	597 (809)	294 (363)	**<0.001**

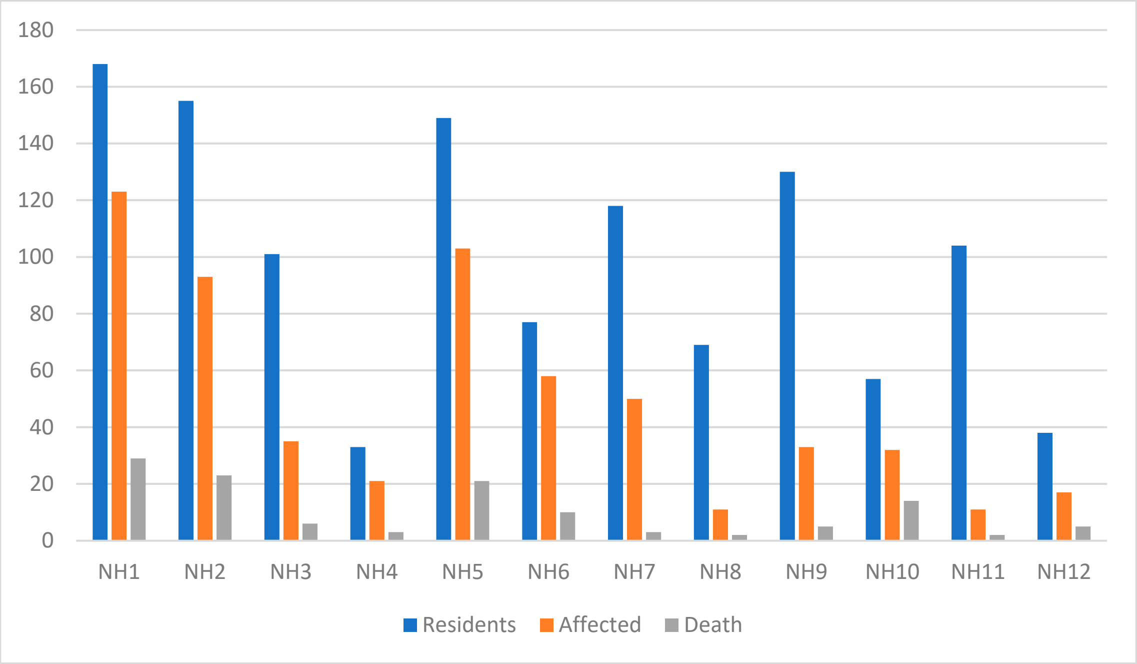

Figure S1: Distribution of residents in all the nursing homes.
